# Evaluation of ELISA and haemagglutination inhibition as screening tests in serosurveillance for H5/H7 avian influenza in commercial chicken flocks

**DOI:** 10.1017/S0950268817002898

**Published:** 2018-01-12

**Authors:** M. E. Arnold, M. J. Slomka, A. C. Breed, C. K. Hjulsager, S. Pritz-Verschuren, S. Venema-Kemper, R. J. Bouwstra, R. Trebbien, S. Zohari, V. Ceeraz, L. E. Larsen, R. J. Manvell, G. Koch, I. H. Brown

**Affiliations:** 1 Department of Epidemiological Sciences (Biomathematics and Risk Research), Animal and Plant Health Agency (APHA), The Elms, College Road, Sutton Bonington, Loughborough LE12 5RB, UK; 2 European Union Reference Laboratory for Avian Influenza and Newcastle Disease, APHA-Weybridge, Woodham Lane, Addlestone KT15 3NB, UK; 3 Avian Virology Workgroup, Virology Department, APHA-Weybridge, Woodham Lane, Addlestone KT15 3NB, UK; 4 Department of Epidemiological Sciences (Epidemiology), APHA, Kendal Road, Harlescott, Shrewsbury SY1 4HD, UK; 5 National Veterinary Institute, Technical University of Denmark (DTU), Kemitorvet, Building 204, 2800 Lyngby, Denmark; 6 Wageningen Bioveterinary Research, Houtribweg 39, 8221 RA Lelystad, The Netherlands; 7 National Veterinary Institute (SVA), Ulls väg 2B, Uppsala, Sweden

**Keywords:** AIV (avian influenza virus), Bayesian, H5/H7 AIV surveillance, ELISA (enzyme-linked immunosorbent assay), HI (haemagglutination inhibition)

## Abstract

Avian influenza virus (AIV) subtypes H5 and H7 can infect poultry causing low pathogenicity (LP) AI, but these LPAIVs may mutate to highly pathogenic AIV in chickens or turkeys causing high mortality, hence H5/H7 subtypes demand statutory intervention. Serological surveillance in the European Union provides evidence of H5/H7 AIV exposure in apparently healthy poultry. To identify the most sensitive screening method as the first step in an algorithm to provide evidence of H5/H7 AIV infection, the standard approach of H5/H7 antibody testing by haemagglutination inhibition (HI) was compared with an ELISA, which detects antibodies to all subtypes. Sera (*n* = 1055) from 74 commercial chicken flocks were tested by both methods. A Bayesian approach served to estimate diagnostic test sensitivities and specificities, without assuming any ‘gold standard’. Sensitivity and specificity of the ELISA was 97% and 99.8%, and for H5/H7 HI 43% and 99.8%, respectively, although H5/H7 HI sensitivity varied considerably between infected flocks. ELISA therefore provides superior sensitivity for the screening of chicken flocks as part of an algorithm, which subsequently utilises H5/H7 HI to identify infection by these two subtypes. With the calculated sensitivity and specificity, testing nine sera per flock is sufficient to detect a flock seroprevalence of 30% with 95% probability.

## Introduction

Avian influenza virus (AIV) subtyping is based on their haemagglutinin (HA) and neuraminidase (NA) envelope glycoproteins, with currently 16 antigenically different HA (H1–H16) and nine NA (N1–N9) subtypes [1]. Most commonly, AIV introductions from wild birds into poultry result in few (if any) obvious clinical signs, and are termed low pathogenicity (LP)AI [2]. However, H5 and H7 AIV subtypes have the potential to mutate to highly pathogenic (HP) AIVs in Galliforme poultry such as chickens and turkeys, resulting in high mortality [3]. Consequently, all H5 and H7 AIV poultry incursions require statutory interventions to prevent spread and control outbreaks [4–6], with H5 and H7 AIVs of both pathotypes considered as notifiable pathogens [7]. In addition, H5 and H7 influenza A viruses include H5N1 HPAIV and H7N9 LPAIV as proven zoonotic pathogens [8].

The presence of clinical signs in Galliformes enabled HPAI to be detected early in most recent instances in Europe, through passive surveillance [9]. Many H5/H7 LPAI outbreaks [10, 11] originate via LPAIV incursion into poultry from wild birds and then present a risk for evolution into the HP phenotype [1]. Because LPAIV infections in poultry normally proceed sub-clinically or with minor signs [12], serological monitoring for H5/H7 AIVs is indicated [13]. There are precedents of prolonged H5/H7 LPAIV dissemination, some of which include subsequent mutation to the corresponding HPAIV [14–16]. Extensive H7N1 LPAIV spread in Italy during 1999 preceded an extensive H7N1 HPAIV outbreak, and contributed to the introduction of mandatory European Union (EU) poultry surveillance [13], with the presence of H5/H7 subtype-specific antibodies identifying flocks infected with these AIV subtypes. The flocks are then investigated further for possible ongoing infection [13]. National poultry surveillance aims to reduce the risk of subsequent HPAI emergence, and provides an epidemiological indication of whether H5/H7 subtypes may be circulating in a particular sector of the EU poultry industry. Freedom from infection is also important to facilitate trade [4]. A range of diverse poultry production practices with perceived risks of H5/H7 AIV incursions is considered in different EU countries. These differences influence the extent of flock sampling in national surveillance programmes [17]. The results are published annually [18, 19].

From the laboratory perspective of carrying-out poultry surveillance, the haemagglutination inhibition (HI) test is a well-established assay that has the advantage of detecting haemagglutinin (HA) type-specific antibodies [20]. Therefore, H5 and H7 HI testing is recommended for the identification of past infection with these subtypes [7, 21]. Whole virus, live or inactivated, is used as antigen for the HI test. The EU testing algorithm recommends defined H5 and H7 viruses as the primary broadly reactive HI antigens for the screening of H5 and H7 antibodies [13]. Sera from flocks that are positive in the primary H5/H7 HI test are then retested by using other H5 or H7 antigens with a different NA subtype, these being the defined secondary HI antigens. Confirmation of H5/H7 infection by HI with the corresponding secondary antigen is important to exclude the possibility that the primary HI-positive result was influenced by cross-reactivity with the NA subtype [22]. The EU-recommended primary and secondary H5 and H7 HI antigens have broad overlapping serological reactivity to contemporary H5 and H7 AIVs circulating in Europe. All H5/H7 confirmed serological findings must be followed-up by testing additional sera and swabs collected from the poultry [13]. Testing of the former may reveal increasing seroconversion in instances of a very recent incursion, while testing of the latter by virus isolation (VI) or AIV reverse transcription Real Time (RRT)–PCR may confirm or exclude ongoing virus circulation at the premises [23].

This study assessed an alternative approach using a commercial blocking ELISA for the initial screening of poultry. This assay detects antibodies directed against the conserved nucleoprotein (NP), thereby detecting humoral responses to all AIV subtypes [7]. To identify evidence of H5/H7 LPAIV infection, all ELISA-positive sera were subsequently tested in the HI test by using both primary H5 and H7 antigens. Sera that return a positive result with the initial HI test with either primary H5 or H7 antigens are then retested using secondary H5 or H7 antigen to confirm past H5/H7 AIV infection [13]. To evaluate the suitability of this new testing algorithm to identify H5/H7 infection, the sensitivity and specificity of both the ELISA and the primary H5/H7 HI tests were determined and compared. While HI is a long-established test for the detection of HA subtype-specific antibody, which continues to be recommended, it cannot be uncritically assumed to represent a ‘gold standard’, particularly when the current OIE test validation guidelines are considered [24]. It is imperative that the screening approach has a proven high sensitivity to prevent false negative results. This study determined the sensitivity and specificity of the two screening methods by means of Bayesian mathematical modelling which, importantly, does not presuppose that either ELISA or primary H5/H7 HI is a ‘gold standard’ to which the other is compared.

## Methods

### Sera

Chicken sera (1055) from 74 commercial farms were tested in this study. All were sourced from a subset collected for the national poultry surveillance programmes in four EU Member States (MSs) (namely Denmark, Sweden, UK and The Netherlands) during the years 2009 (one flock in Denmark, 39 sera) and 2012–2013 for the remaining 73 flocks in Sweden (40 sera, four flocks), the UK (245 sera, 17 flocks), The Netherlands (284 sera, 11 flocks) and Denmark (447 sera, 41 flocks) (Table 1). The 17 UK chicken flocks, all AIV seronegative, were selected randomly during national poultry surveillance, as were 40 of the 42 Danish flocks, four of which were seropositive for non-H5/H7 AIV. However, non-random inclusion of H5/H7 and non-H5/H7 seropositive flocks was required to provide sufficient data for the Bayesian model. Two additional Danish flocks were therefore deliberately included to provide H5- and H7-positive sera (Table 2). The intense AI poultry surveillance in The Netherlands also provided 11 of the 21 AIV seropositive chicken flocks in this study (Tables 1 and 2). Therefore The Netherlands flocks were also not selected randomly. The 11 H5/H7 seropositive flocks identified in Denmark and The Netherlands provided the majority of the chicken sera, which were positive by either the primary H5/H7 HI and/or ELISA (*n* = 282; Table 2). Sera from these 11 flocks were critical to enabling the model to determine the sensitivity and specificity of screening for H5/H7. Secondary HI testing confirmed all 11 H5/H7 seropositive flocks. The four Swedish flocks which were seropositive for non-H5/H7 were also selected non-randomly (Table 2). The non-random selection of chicken flocks was factored into the Bayesian model.

**Table 1.** Description of the 74 chicken flocks and flock-level serology results

Country	Layers	Outdoor layers	Organic layers	Broilers	Breeders	Total flocks sampled per country	Total sera	AI positive flocks	H5/H7- positive flocks
The Netherlands		7	4			11	284	11	9
Denmark	6	7	19	5	5	42	486	6	2
Sweden			2	1	1	4	40	4	0
UK	14				3	17	245	0	0
Totals	20	14	25	6	9	74	1055	21	11

Total numbers of chicken flocks and sera tested by primary H5/H7 HI and ELISA in each of the four countries. At the flock-level, flocks are categorised as either AI or H5/H7 positive. EU definitions of free-range poultry as either ‘outdoor’ or ‘organic’ are summarised at: http://www.britishpoultry.org.uk/introduction-to-marketing-standards-for-free-range-and-organic-poultry-meat/ (Accessed 13 October 2017).

The six Danish layer flocks consisted of one caged-layer flock and five layer-breeder flocks. The breeder flocks from Denmark (*n* = 5) and Sweden (*n* = 1) were all broilers. Further details were unavailable for the UK outdoor layer (*n* = 14) and breeder (*n* = 3) flocks.

**Table 2.** Serology results from the 21 AI-positive flocks from The Netherlands, Denmark and Sweden

Flock identity number and HA subtype	Type of chicken flock	ELISA result/primary HI result				Total sera tested per flock	Observed AI seroprevalence per flock	
		Pos/Pos	Pos/Neg	Neg/Pos	Neg/Neg		ELISA (%)	Primary HI (%)
The Netherlands								
1 H7	Organic layer	4	6	0	8	18	55.6	22.2
2 H5	Outdoor layer	3	13	0	9	25	64	12
3 H5	Outdoor layer	13	8	1	16	38	55.3	36.8
4 H7	Outdoor layer	7	7	0	15	29	48.3	24.1
5 H5	Outdoor layer	2	19	0	9	30	70	6.7
6 H7*	Organic layer	16	6	0	8	30	73.3	53.3
7 H7	Outdoor layer	15	3	0	0	18	100	83.3
8 H6*	Organic layer	0	15	0	15	30	50	0
9 H5	Organic layer	2	19	0	8	29	72.4	6.9
10 Non-H5/H7	Outdoor layer	0	16	0	14	30	53.3	0
11 H5	Outdoor layer	1	6	0	0	7	100	14.9
Denmark								
12 Non-H5/H7	Outdoor layer	0	1	0	9	10	10	0
13 Non-H5/H7	Organic layer	0	1	0	9	10	10	0
14 Non-H5/H7	Organic layer	0	9	0	1	10	90	0
15 Non-H5/H7	Organic layer	0	9	0	1	10	90	0
16 H7	Organic layer	10	28	0	1	39	97.4	25.6
17 H5	Outdoor layer	15	2	1	1	19	89.5	84.2
Sweden								
18 Non-H5/H7	Organic layer	0	9	0	1	10	90	0
19 Non-H5/H7	Organic layer	0	1	0	9	10	10	0
20 Non-H5/H7	Broiler	0	3	0	7	10	30	0
21 Non-H5/H7	Broiler-breeder	0	1	0	9	10	10	0
Total sera		88	182	2	150	422		

All sera were tested by both ELISA and primary H5/H7 HI. The four central columns indicating the four results categories present the data which were analysed by the Bayesian model. All H5 and H7 seropositive flocks were confirmed by testing with the corresponding secondary HI antigen. * Indicates H7N7 LPAIV isolation from flock 6 in The Netherlands, while H6 seropositive flock 8 was identified by additional HI testing with non-H5/H7 antigens (data not shown).

### HI antigen preparation

Four HI viral antigens of the H5 and H7 subtypes were grown for surveillance purposes in 9–11-day-old embryonated fowls’ eggs at the Animal and Plant Health Agency (APHA, UK) using standard protocols [7, 21]. Allantoic fluid harvests were inactivated by adding neat beta-propiolactone (Ferak, Berlin, Germany) as a 1 : 2000 dilution which produced an 8 mM final strength solution. The mixture was incubated for 16 h at 37 °C with stirring. One ml volumes of allantoic fluid were freeze-dried and distributed to the participating laboratories, for reconstitution in 1 ml sterile distilled water. The primary H5 and H7 HI antigens were A/teal/England/7394-2805/06 (H5N3) and A/turkey/England/647/77 (H7N7), respectively, while the secondary HI antigens were A/chicken/Scotland/59 (H5N1) and A/African starling/England/983/79 (H7N1) [13].

### Serological testing

The chicken sera collected from a given MS were tested at the respective AI National Reference Laboratory (NRL). All sera were tested by the multi-species influenza A ELISA (IDEXX, France) according to the manufacturer's instructions. This blocking ELISA detects antibodies against the influenza A NP antigen, which is common to all subtypes. All sera were also tested by HI using the primary H5 and H7 antigens by using four HA units per test and a cut-off where a serum dilution titre of 1 : 16 or greater was considered positive [7, 13, 21]. All sera from flocks which registered positive primary HI reactivity in at least one serum were retested by the corresponding secondary H5 or H7 HI antigens to confirm seropositivity for H5/H7. The H5/H7 HI testing was restricted to the use of the surveillance (heterologous) antigens, which reflected normal practice during the EU national poultry surveillance programmes [13]. One flock in The Netherlands was identified as having been actively infected with an H7N7 LPAIV at the time of serological sampling of chickens (Table 2), but no H7 HI testing was done with the homologous H7 LPAIV isolated from this flock. All four AI NRLs involved in this study participate in annual HI proficiency testing to demonstrate their mandatory test competence [25].

### Statistical methods

A Bayesian method was employed to estimate the sensitivity and specificity of the ELISA and primary H5/H7 HI tests, and the true within-flock seroprevalence. Bayesian methods have been shown to be highly useful for diagnostic test evaluation in the absence of a gold standard [26, 27]. The key feature of a Bayesian method is that prior assumptions for all the unknown parameters are combined with maximum-likelihood inference on the available data to provide final (posterior) estimates, which are a weighted average of the prior assumptions and inference from the data. In the present study, we chose to have non-informative priors for the test sensitivities, specificities and within-flock prevalence (beta distributions with both parameters equal to 1, which are uniform in the range 0–1) so that the final outputs were totally driven by the study data. The design of the Bayesian model assumed that the sensitivity of the primary H5 and primary H7 HI tests was the same. The analysis considers both primary H5/H7 HI tests as a screening test which, when followed by secondary HI testing, leads towards the identification and confirmation of past H5/H7 infection. The model was not based on random testing of flocks in all of the countries, but was designed to analyse input data consisting of the numbers of sera belonging to each of four categories of test results in each flock. These were the number of results which were: (1) positive by both ELISA and primary HI, (2) positive by ELISA but negative by primary HI, (3) negative by ELISA but positive by primary HI and (4) negative by both tests (Table 2).

Let the within-flock seroprevalence of H5/H7 be denoted by *π*. For the data from The Netherlands, intensive efforts were made to establish the strain type, and there was no evidence of multiple strains infecting any of the flocks. In addition, the generally good standards of biosecurity practiced by the commercial poultry sector in Europe and history of results from recent years’ EU poultry surveillance [18, 19] allowed the assumption to be made that the chickens had been exposed to only one strain of AIV, i.e. we assumed that the chickens will not have been infected by both H5/H7 and non-H5/H7 AIVs. The sensitivity and specificity of the primary HI to detect H5/H7 AI are denoted by *Se*_*H*_, *Sp*_*H*_, and the sensitivity and specificity of the ELISA to detect avian influenza of any subtype is denoted as *Se*_*E*_, *Sp*_*E*_. We assumed that there was no difference in the sensitivity and specificity of ELISA according to the subtype, so that it would detect H5/H7 and non-H5/H7 antibodies with the same likelihood.

Flocks that were positive only for ELISA were excluded from the Bayesian model, since these flocks provided no information on the sensitivity of primary H5/H7 HI, and it would also require a more complex model to incorporate this data into the model for the little extra benefit. However, the seroprevalence was estimated for these flocks by taking into account the sensitivity and specificity of ELISA estimated from the HI and ELISA-positive flocks, and the flocks which were negative for both HI and ELISA.

With these assumptions, the likelihood of both primary HI and ELISA testing positive on a serum sample from any bird within a flock is given by the sum of (i) the probability that the bird had antibodies to H5/H7 AIV and correctly identified by both tests, *πSe*_*H*_*Se*_*E*_, and (ii) the probability that the bird did not have antibodies to H5/H7 AIV and was incorrectly classified by both tests (1 − *π*)(1 − *Sp*_*H*_)(1 − *Sp*_*E*_). Similar reasoning can be applied to derive the probabilities for the other test outcomes. Denoting by *p*_*ij*_, *i*, *j* = 0, 1 the likelihood that primary HI or ELISA was negative (*i*,*j* = 0, respectively) or positive (*i*,*j* = 1), respectively, the other test outcomes had the following probability:

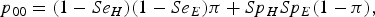


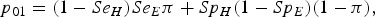


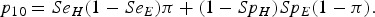


The outcome for each bird in each flock arises from a multinomial distribution with the probability of each of the four possible outcomes given by*p*_*ij*_. The basic idea aim of the study is to apply statistical approaches to find the values of the sensitivity and specificity of each test and the within-flock prevalence that produces the best fit to the observed data, i.e. effectively solving equations *p*_00_, *p*_01_, *p*_10_, *p*_11_ for each flock using a Bayesian approach. The multinomial model was fitted to the data from each flock using WinBUGS 3.1. The approach works by having initial starting values, which converge towards the final (posterior) estimates and so the first 5000 iterations were discarded as a burn-in period before convergence had been reached. After the burn-in period, 10 000 iterations were used to generate the final (posterior) estimates, and convergence assessed using the Gelman–Rubin statistic, as implemented in WinBUGS.

The fit of each model to the data was assessed by the use of Bayesian *P*-values, which are a measure of model fit based on Pearson's chi-squared statistics [28], where a low *P*-value represents a poor fit of the model to the data. The WinBUGS code for the model was adapted from a previously supplied code [29].

### Calculation of sample sizes for screening

The number of serum samples required to detect exposure to H5/H7 AIV using primary HI or ELISA at the initial screening was calculated according to the following formula, based on the formula for sampling large flocks/herds [30]:

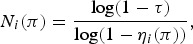

where *τ* is the required statistical power. For the estimate of the sensitivity of the method given the within-flock prevalence (*η*_*i*_(*π*)), the median estimate of the Bayesian posterior was used. Sample sizes were estimated for each sample type for the detection of the following within-flock infection seroprevalences: 2.5%, 5%, 10%, 20%, 30%, 40% and 50%.

## Results

### Serological results from commercial chickens

All 245 sera collected from 17 chicken flocks in the UK were negative by both the ELISA and both primary HI antigens (Table 1). The remaining 57 flocks tested in the three other countries included 21 AI seropositive flocks. Among these, there were six flocks seropositive for H5 and five seropositive for H7. Nine of these seropositive flocks occurred in The Netherlands and two in Denmark (Tables 1 and 2). The 11 H5/H7 seropositive flocks were confirmed by retesting with the corresponding H5 or H7 secondary HI antigen (data not shown). In The Netherlands, H7N7 LPAIV was successfully isolated from one H7 seropositive flock to indicate active ongoing infection, while one non-H5/H7 infected flock was characterised as being H6 seropositive by additional testing (Table 2).

### Estimation of test sensitivity and specificity by applying the Bayesian model

Within the 11 H5/H7-positive flocks, there were 205 sera that were ELISA positive and 90 that were primary H5/H7 HI-positive sera. Two sera were negative by ELISA and positive by primary HI compared with 117 sera that were positive for ELISA and negative by primary HI (Table 2). Consequently, the model estimates of ELISA sensitivity (97%) are much higher than that of primary HI (43%, Table 3). High specificity (>99%) was determined for both primary HI and ELISA (Table 3).

**Table 3.** Sensitivity and specificity estimates (Bayesian) of the ELISA and primary H5/H7 HI tests

Parameter	Description	Bayesian estimates	
		Median	2.5 and 97.5 percentiles
*SeH*	Sensitivity of HI	0.43	(0.36, 0.50)
*SpH*	Specificity of HI	0.998	(0.994, 1.0)
*SeE*	Sensitivity of ELISA	0.97	(0.94, 0.99)
*SpE*	Specificity of ELISA	0.998	(0.993, 1.0)

**Fig. 1.**
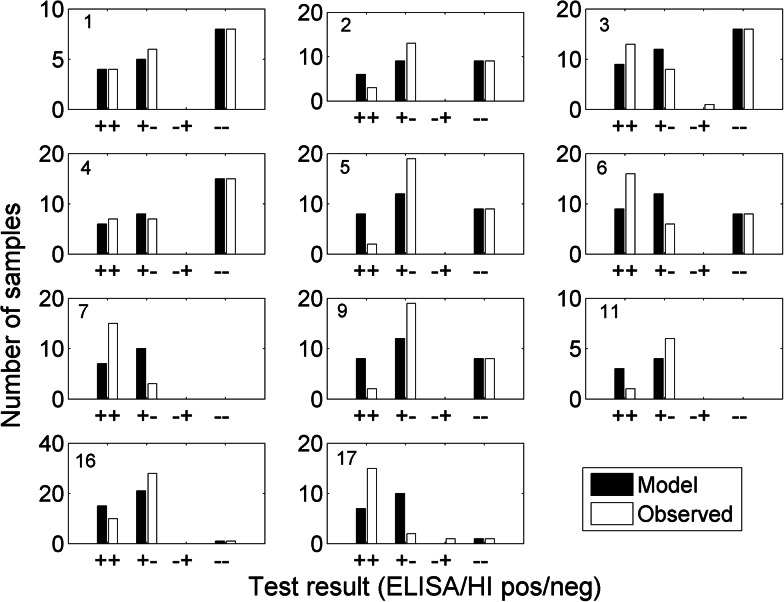
The fit of the Bayesian model (black bars) to the observed data (white bars) for each of 11 H5/H7 AI-positive flocks tested with both ELISA and primary H5/H7 HI in The Netherlands and Denmark. The 11 flock identifiers correspond to those listed in Table 2.

While there was no evidence of an unsatisfactory fit to the model overall, with a Bayesian *P*-value of 0.11 for the model fit to the dataset as a whole, there was an apparent large variability in the sensitivity of the primary HI between flocks. For example, in flocks 5 and 9, the model significantly overestimates the number of HI positives, suggesting a much lower sensitivity than 43% for primary HI in those flocks (also evidenced by the lower relative sensitivity of HI to ELISA: 2/21 (9.5%) observed in both flocks; Fig. 1). In flocks 7 and 17, the model greatly underestimates the number of HI positives, suggesting a much higher sensitivity in those flocks (relative sensitivity of HI to ELISA 15/18 (83.3%) and 15/17 (88.2%) observed in flocks 7 and 17, respectively; Fig. 1).

### Sample size estimation

Because of the lower sensitivity of the primary HI test compared with the ELISA, much larger sample sizes were required when using the HI test, compared with the ELISA, to achieve the same level of confidence in determining a flock's status. Using the ELISA, a sample size of nine sera is sufficient to provide 95% confidence of detecting H5/H7 infection in a flock when the seroprevalence exceeds 30%, whereas a sample size of 22 is necessary to achieve the same level of confidence when using HI (Table 4).

**Table 4.** Sample size estimates per flock.

Serological test	Within-flock prevalence (%)						
	2.5	5	10	20	30	40	50
Primary H5/H7 HI	235	127	66	33	22	16	13
ELISA	113	58	29	14	9	7	5

The number of samples required to detect H5/H7 seropositive chickens with a 95% probability for a range of values of within-flock seroprevalence.

### Within-flock seroprevalence

**Fig. 2.**
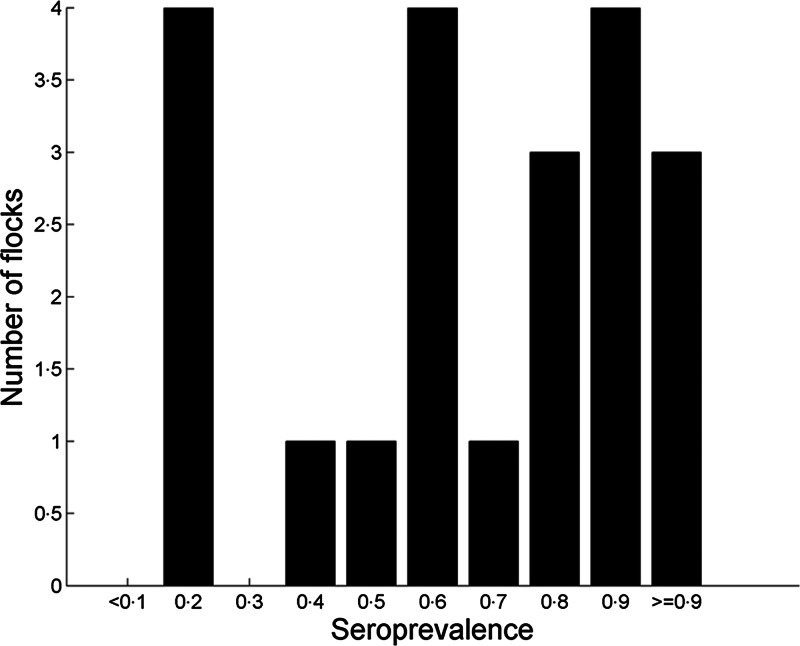
Distribution of the seroprevalence estimates for the 21 AI seropositive flocks from The Netherlands, Denmark and Sweden. Estimates were derived from the application of the Bayesian model to the data obtained from the testing of all chicken flocks by primary HI and ELISA in the study. The actually observed seroprevalences determined by the two tests are listed in Table 2.

The model was able to estimate the seroprevalences in the 21 AIV-infected chicken flocks (Fig. 2), which showed that within-flock prevalence was <50% for the majority of flocks in the study.

## Discussion

The key outcome is that ELISA detection of influenza A antibody is a reliable method for serological screening to seek evidence of H5/H7 LPAIV infection in chicken flocks, with high sensitivity (97.0%) and specificity (99.8%) clearly apparent (Table 3). Screening by primary H5/H7 HI was also highly specific (99.8%), but sensitivity was much lower (43.0%) (Table 3). The two tests differ in detecting antibody responses to different AIV antigens, namely HA and NP, which demonstrate (past) infection by H5/H7 LPAIVs and those caused by all AIV subtypes, respectively. This difference between the tests was considered in the design of the Bayesian model which compared the two tests as the screening step towards identifying H5/H7 AIV infection. The sensitivity and specificity findings show that a surveillance algorithm based on initial screening by the pan-subtype ELISA followed by the H5/H7 HI testing using the heterologous antigens is a potentially preferable approach to identify evidence for H5/H7 AIV infection.

The manufacturer of the ELISA quotes similar high sensitivity (95.4%) and specificity (99.3%) respectively (http://www.idexx.co.uk/pdf/en_gb/livestock-poultry/influenza-a-ab-test-sheet.pdf), but these were derived by a traditional approach where the ELISA was compared relative to an incompletely defined ‘AI serological status’ in avians which served as a ‘gold standard’. The HI test has been long used to measure type-specific humoral responses to natural influenza A virus infection and vaccination, before the emergence of ELISA technology [31]. HI is recommended for poultry surveillance through detection of H5- and H7-specific antibodies [13]. The availability of commercial ELISAs for generic AIV antibody detection has been noted by the OIE, but without any recommendations regarding their use in surveillance [7]. An important aspect of this study was that Bayesian modelling was not dependent on either of the tests being assumed to be a ‘gold standard’, and can robustly estimate the uncertainty in each of the test characteristics (Table 3), which would be more problematic when making such an assumption. This approach is now accepted by the OIE as a valid means of determining test sensitivity and specificity [24].

There was some non-random selection of flocks in order to increase the numbers of H7/H7-positive chickens. If there was a bias caused through a selection of known H5/H7 positives, then it would be more likely to affect the sensitivity estimates; the specificity is largely determined by the 53 negative flocks from the UK and Denmark which were selected randomly.

An advantage of the HI test is that it requires reagents, which are relatively inexpensive to produce by international AI reference laboratories (http://www.oie.int/our-scientific-expertise/reference-laboratories/list-of-laboratories/), although strain selection for primary and secondary H5/H7 HI antigens requires careful consideration for surveillance purposes in a given region, e.g. Europe [13]. The potential confounder of the NA subtype causing HA cross-reactivity has been long known [32], hence the importance of confirmatory secondary HI testing with the same HA subtype but different NA subtype [13, 22]. False-positive results by the H5/H7 primary HI were excluded in the sera tested in this study by virtue of confirmation by the secondary H5/H7 HI testing, and contributed to the high specificity for primary H5/H7 HI. Although it was not possible to entirely exclude the possibility that the poultry flocks may have been seropositive due to earlier infection by a different LPAIV subtype, the model assumed that chickens in the 11 H5/H7 LPAIV-infected flocks had been exposed to only these HA subtypes. This assumption was substantiated by confirmation of all 11 H5/H7-infected flocks as being H5/H7 seropositive by the secondary HI testing. Hence, there was no evidence of prior infection with different HA subtypes that also possessed the N3 or N7 subtypes. The assumption was considered reasonable in view of the high standard of biosecurity in the European commercial poultry sector, in particular chickens, which remains apparent from the annual results of the EU national poultry surveillance programme [18, 19].

The model estimate of ELISA specificity refers to the probability of an AI seronegative bird testing positive. However, the objective of serosurveillance is to detect H5/H7 seropositives, therefore the non-H5/H7-infected birds that would test positive for ELISA (and H5/H7 HI negative) could be viewed as false positives. In this regard, the ELISA will have lower than 99.8% specificity to detect H5/H7 infections. The probability of non-H5/H7 infected birds testing positive for ELISA will depend upon the prevalence of non-H5/H7 strains in the epidemiological setting where the ELISA is applied: any cost-benefit of simpler ELISA screening may be compromised by excessive subsequent H5/H7 HI testing to exclude many infections due to non-H5/H7 AIVs.

Despite the established use of H5/H7 HI in AI laboratories, there are concerns, which reflect the well-known antigenic variability (drift) within a given HA subtype [33, 34]. Such intra-subtype variation may compromise the sensitivity of the primary H5/H7 HI antigens at the screening stage. Considerable variability in the sensitivity of HI relative to the ELISA was observed between flocks, as illustrated by the over/underestimation of HI positives by the model compared with the numbers which were observed (Fig. 1 and Table 2). Reduced sensitivity in some flocks may be caused by a compromised immunogenic fit between the primary H5/H7 HI antigens and the antibody response elicited by the HA of the particular H5 or H7 AIV strains responsible for infection in these flocks. Relatively high sensitivity could imply a stronger HI antigen–antibody relationship. Consequently, the use of an ELISA, which detects humoral responses to the more conserved NP antigen, was considered as an alternative to screening by primary H5/H7 HI. These ELISA reactor flocks require subsequent primary H5/H7 HI testing to identify LPAIV infection with these subtypes.

The heterologous H5/H7 HI antigens selected for surveillance in the EU have been chosen on the basis of broad antigenic reactivity to the corresponding subtypes known to be circulating in Europe during preceding years [13]. However, mandatory poultry surveillance has led to the direct discovery of an ongoing LPAIV outbreak in only a minority of H5/H7 seropositive flocks. For example, during 2015 serological surveillance was done in 21 867 flocks (all poultry species) in the 28 EU MSs, revealing 33 and seven H5 and H7 seropositive holdings, respectively [19]. These 40 flocks included only eight and three premises where active outbreaks of H5 and H7 LPAIV respectively were occurring, as proven by virological investigations. Successful VI provides the homologous HI antigen for any subsequent LPAIV outbreak-related surveillance, but this is not achieved for the majority of H5/H7 seropositive premises. Resolution of the LPAIV incursion and its immunological clearance is the likely explanation in many H5/H7 seropositive flocks.

The availability of blocking ELISAs for detection of type-common AIV antibodies offers benefits for rapid high-throughput screening of poultry flocks. This study showed that the blocking ELISA clearly has superior sensitivity compared with primary H5/H7 HI as the initial screening method for H5/H7 surveillance in commercial chickens. Testing of 102 commercial layer farms (1820 chicken sera) in New Zealand by an indirect ELISA identified 24 AIV seropositive flocks, with only three free-range farms each including a single reactor serum that was H5 HI positive [35]. It was noted that 19 of the 21 AIV seropositive flocks detected by ELISA were free-range layers (Table 2). The blocking ELISA used in the current study has an additional advantage in that it is not dependent on an anti-species conjugate which is a feature of indirect ELISAs, therefore the blocking ELISA may be used to similarly test sera from other poultry species.

Another study has used a Bayesian version of latent class analysis to review and analyse previously published data to compare HI and four different in-house ELISAs. It concluded that HI had a higher diagnostic sensitivity [36]. However, that study used a different methodology where sera were drawn from the field- and experimentally infected chickens during the acute post-infection stage and used the homologous (or a near homologous) HI antigen. This resulted in the HI having higher diagnostic sensitivity than the ELISAs [36]. HI testing with the homologous H5/H7 antigens did not feature in our study, this reflecting the commonly encountered scenario whereby few H5/H7 seropositive flocks identified by EU poultry surveillance successfully yield the homologous antigen through VI. Use of the heterologous EU-recommended H5/H7 HI antigens potentially reduced the sensitivity of the HI testing because of the unknown degree of immunogenic diversity within the H5/H7 subtypes.

Temporal effects may also influence the results of antibody testing. It is accepted that any test for AIV antibody may remain potentially vulnerable to false negative results if the collection of sera is confined to a very early window prior to seroconversion. H7 LPAIV chicken infection studies showed seroconversion to occur between 1 and 2 weeks post-infection (e.g. [37]). These results were obtained by using another anti-NP blocking ELISA, with accompanying homologous H7 HI being more sensitive than the heterologous surveillance H7 HI antigens employed in the current study. In an H5/H7 LPAIV field setting, however, not all chickens would be at the same stage of infection, with some birds already seroconverting while others are at the earlier stage of initial viral shedding. In the event of any detectable seropositivity, the EU recommends a prompt epidemiological investigation to include follow-up sampling of poultry to include swabs for virological investigation plus subsequent sera to identify any seroprevalence increase during acute stage seroconversion [13]. Relatively little is known concerning the decline of AIV antibodies in experimentally infected chickens at later time points, although one study used homologous H7 HI to demonstrate a decline in HI titres 8 weeks post-infection [38]. To our knowledge, there is no published prolonged chicken infection study which specifically used an anti-NP blocking ELISA. However, it is also possible that lower seroprevalences such as those observed in six flocks in the range 10–48.3% (by ELISA, Table 2) may be due to the LPAIV infection not spreading efficiently within the flock.

Current EU guidelines recommend the collection of 10 sera per chicken or turkey flock for H5/H7 AIV serosurveillance, and this would be a sufficient sample size to provide 95% confidence of detecting infection if it is present at the design seroprevalence of 30% [13], if initial screening is done by the blocking ELISA (Table 4). AI seroprevalence of greater or equal to 30% was observed in 17 of the 21 AI-infected flocks (Table 2), so it is possible that at least some of the lower seroprevalence flocks would have been undetected by sampling 10 sera for testing by blocking ELISA. Again, these may represent inefficient intra-flock LPAIV spread or historical LPAIV infections with declining seroprevalence and no active shedding at the time of chicken sampling. Such a historical infection scenario may have occurred at three H5 seropositive layer farms, which were identified in New Zealand at a low seroprevalence (1/10) following type-common ELISA screening and HI confirmation. Follow-up AIV RRT–PCR investigations excluded any ongoing outbreaks [35].

In conclusion, the greater sensitivity of the blocking ELISA has clearly demonstrated its preferred use for screening during serological surveillance for H5/H7 infection in EU commercial chickens. Serological surveillance is therefore not applied primarily as an early warning system for direct detection of ongoing AIV outbreaks in individual flocks, but provides evidence for prior exposure and circulating AIV within poultry populations from different geographical regions, poultry species and management types.
